# Analysis of Microbial Diversity and Dynamics During Bacon Storage Inoculated With Potential Spoilage Bacteria by High-Throughput Sequencing

**DOI:** 10.3389/fmicb.2021.713513

**Published:** 2021-09-28

**Authors:** Xinfu Li, Qiang Xiong, Hui Zhou, Baocai Xu, Yun Sun

**Affiliations:** ^1^College of Food Science and Light Industry, Nanjing Tech University, Nanjing, China; ^2^School of Food Science and Biology Engineering, Hefei University of Technology, Hefei, China

**Keywords:** microbial diversity, bacon, spoilage bacteria, high-throughput sequencing, storage

## Abstract

*Staphylococcus xylosus, Leuconostoc mesenteroides, Carnobacterium maltaromaticum*, *Leuconostoc gelidum*, and *Serratia liquefaciens* were investigated for their roles in in the spoilage of sterilized smoked bacon. These five strains, individually and in combination, were applied as starters on sliced bacon at 4–5 log_10_ CFU/g using a hand-operated spraying bottle and stored for 45 days at 0–4°C. Dynamics, diversity, and succession of microbial community during storage of samples were studied by high-throughput sequencing (HTS) of the V3–V4 region of the 16S rRNA gene. A total of 367 bacterial genera belonging to 21 phyla were identified. Bacterial counts in all the inoculated specimens increased significantly within the first 15 days while the microbiota developed into more similar communities with increasing storage time. At the end of the storage time, the highest abundance of *Serratia* (96.46%) was found in samples inoculated with *S. liquefaciens*. Similarly, for samples inoculated with *C. maltaromaticum* and *L. mesenteroides*, a sharp increase in *Carnobacterium* and *Leuconostoc* abundance was observed as they reached a maximum relative abundance of 97.95 and 81.6%, respectively. Hence, these species were not only the predominant ones but could also have been the more competitive ones, potentially inhibiting the growth of other microorganisms. By analyzing the bacterial load of meat products using the SSO model, the relationships between the microbial communities involved in spoilage can be understood to assist further research.

## Introduction

Bacon is widely consumed in Europe, North America, and some other parts of the world (Soladoye et al., 2015) but since they are highly susceptible to microbial contamination, thermal processing can be applied to reduce the bacterial load of meat products. However, some strains are still able to resist this heat-processing step (Li et al., 2021). For example, one report found that during refrigerated storage, microorganisms, such as *Leuconostoc carnosum* or *Weissella viridescens* survived, resulting in post-heat treatment recontamination and eventually, in spoilage (Zagdoun et al., 2020). The spoilage of cooked and cured meat is generally considered to be the result of the growth and reproduction of microbes, such as *Leuconostoc* spp., *Lactobacillus* spp., *Enterobacteriaceae*, *Carnobacterium* spp., *Pseudomonas*, and *Brochothrix thermosphacta* (Borch et al., 1996; Korkeala and Björkroth, 1997; Samelis et al., 2000; Nychas et al., 2008) which, collectively, may be known as “specific/ephemeral spoilage microorganisms-S(E)SO,” as displayed in Table 1. Previous research indicates that these microorganisms dominate the meat matrix and produce spoilage-associated changes, such as slime, -odors, and other undesirable flavors (Nychas et al., 2008; Casaburi et al., 2015).

**TABLE 1:** SSO/Dominant/Starter organism and the technique used in their determination in meat and meat products.

SSO/Dominant/Starter organism	Product	Technique	References
*Leuconostoc mesenteroides*, *Leuconostoc carnosum*, *Leuconostoc gelidum*, *Lactobacillus sakei*	Cooked ham, cooked meats, vacuum-packaged beef, bacon, beef	PFGE, culture-dependent, PCR, 16S rRNA sequencing	Korkeala and Björkroth, 1997; Björkroth et al., 1998; Samelis et al., 2000; Metaxopoulos et al., 2002; Hamasaki et al., 2003; Chenoll et al., 2007; Säde, 2011; Pothakos et al., 2014b; Pothakos et al., 2015; Comi et al., 2016; Li et al., 2019
*Carnobacterium maltaromaticum*, *Carnobacterium divergens*	Processed meat products, ham, bacon, fermented sausages, chicken	Culture-dependent, PCR, 16S rRNA sequencing	Leisner et al., 1995; Chenoll et al., 2007; Leisner et al., 2007; Casaburi et al., 2011
*Serratia liquefaciens*, *Rahnella aquatilis*, *Hafnia alvei*	Cooked/cured meat products, meat, minced meat, dry-cured ham	Culture-dependent, PCR, 16S rRNA sequencing	Lindberg et al., 1998; Gram et al., 1999; Doulgeraki et al., 2011; Belletti et al., 2013
*Staphylococcus xylosus*	Cured ham, fermented foods, sausage	Culture-dependent, PCR	Kotzekidou and Bloukas, 1996; Paarup et al., 1999; Leroy et al., 2006; Mah and Hwang, 2009; Ravyts et al., 2012

Different microbial taxa/species may be randomly developed during meat storage, thus influencing the type of spoilage development (Ercolini et al., 2009). This is because the spoilage process is a complex event involving biological activities which are likely to be different for different microorganisms. Moreover, details on the species involved in the spoilage of meat are still unclear and needs to be further assessed. Therefore, it is necessary to characterize these organisms, both at the species as well as the biotype levels, in order to better understand the spoilage process. Furthermore, SSOs contribute to spoilage despite having an initial low population (Säde, 2011). Hence, an appropriate method for describing and understanding their growth and activity, or even for evaluating their spoilage potential is also crucial (Pothakos et al., 2014a).

In this context, Pin et al. (1999) as well as McMeekin and Ross (1996) have suggested that the method involving the inoculation of sterile substrates with spoilage organisms provided a more accurate way for representing and predicting the growth of food spoilage organisms by comparing their growth rates. In fact, over the past few years, microbiological growth and spoilage potential of isolated bacterial species have been monitored using this SSO model (Mataragas et al., 2007). For example, many studies reported its application for fish products (Stohr et al., 2001; Macé et al., 2013; Wang et al., 2017), for processed meat containing beef (Leisner et al., 1995; De Filippis et al., 2013) or saveloy (Holm et al., 2013), and even for packaged meat products (Rahkila et al., 2012). Furthermore, this SSO model was also applied to evaluate the ability of some isolates [e.g., lactic acid bacteria (LAB)] to act as protective cultures for bio-preservation (Bredholt et al., 2001; Vermeiren et al., 2004; Alves et al., 2006). However, the microbiological studies reported in the above-mentioned studies were almost carried out using a culture-dependent approach (traditional microbial cultivation). This method can be rather unreliable when trying to provide accurate information about microbial communities in an ecosystem as only a small portion of the true microbial population can be cultivated. As an alternative, culture-independent methods, especially high-throughput sequencing (HTS), has been successfully applied in meat microbiology research to monitor the dynamic changes in microbial flora as this approach can provide more detailed information about the microbial communities compared with other molecular methods. However, to the best of our knowledge, as far as bacon is concerned, only few studies have been conducted so far. Therefore, it would be useful to apply this HTS technology to analyze the growth and activity of spoilage microorganisms in bacon so that a deeper and more precise evaluation of its spoilage process can be made. This study enables us to understand the growth one taxa/species dominates the spoilage and is affected by the others.

In our previous study, *Staphylococcus xylosus*, *Carnobacterium maltaromaticum*, *Leuconostoc mesenteroides*, *Serratia liquefaciens*, and *Leuconostoc gelidum* were identified and considered responsible for the potential spoilage characteristics of bacon (Li et al., 2019). In this work, sterile bacon was inoculated with these five isolated organisms before investigating the bacon’s bacterial diversity using HTS, in order to gain a deeper understanding of the dynamic nature of the microbial population during the spoilage process. Furthermore, changes in the physicochemical properties of the meat were also measured to evaluate how they were connected with the microbiota.

## Materials and Methods

### Bacterial Strains and Sterile Samples

All strains used in this study were selected from the laboratory collection team, were previously isolated and identified from smoked bacon during refrigerated storage (Li et al., 2019), and maintained as frozen stocks at −80°C in a strain storage medium (Qingdao Hope Bio-Technology Co., Ltd., Qingdao, China). These strains belonged to the taxonomic groups: *S. xylosus*, *L. mesenteroides*, *C. maltaromaticum*, *L. gelidum*, *S. liquefaciens*, and combination of the above five strains at the same concentration could be stored in a sterile vial and mixed (Pm).

All vacuum-packaged bacon samples were prepared in a local western-style meat-processing company without use of preservatives. Specimens were approximately 200 mm long, 40 mm wide, and 2.5 mm thick, with eight or nine slices (around 200 g per bag). Sterile bacon was prepared to prevent any influence of bacterial impurities. To avoid the interference from natural microbiota, samples were immediately transferred in insulated boxes containing dry ice and thereafter sterilized by irradiation at a dose of 6 kGy (Dogbevi et al., 1999; Pothakos et al., 2015; Wang et al., 2017) using a ^60^Co source at Hangyu (Hangyu Irradiation Technology Co., Ltd., Nanjing, China). The samples that had been irradiated were immediately transported back to the laboratory, and then stored for about 1 week at −80°C for inoculation.

### Strain Culture, Sample Inoculation, and Packaging

The strains, deep-frozen before use, were reanimated, streaked by a single rolling magnetic bead driven by a magnet on the selective culture media from which the colonies were originally harvested by traditional microbiological methods, the cultures were recovered and incubated at the aforementioned temperature and time (Li et al., 2019). According to the previous method (Roig-Sagués and Eerola, 1997; Stavropoulou et al., 2018), growth of the precultures was verified twice during cultivation, then one standardized loopful of pure colonies was suspended in a tube of MRS broth, brain heart infusion (BHI) broth, and TSB (Triptone Soya Broth, Oxoid) by propagation at 30°C for 24 h. Thereafter, 0.1 mL was removed to another tube including the related broth supplemented and incubated at 30°C for 24 h anaerobically. The cell pellets were accumulated by centrifugation (4,000 rpm, 4°C, 10 min), then thrice-washed and resuspended in 50 mL of Ringer’s solution (Oxoid), thereafter the cellular concentration was adjusted to approximately 6–7 log_10_ CFU/mL as inoculum for the meat preparation. The five strains were added to a sterile vial and then stirred to homogenize the mixture to achieve the desired inoculation rate.

Before analysis, bacon specimens were thawed for 24 h at 0–4°C. A total of 108 samples were randomly divided into seven groups. Next, approximately equal volumes and concentrations of suspensions of strains were sprayed onto the surface of the six grouped samples by using a hand-operated spraying bottle: the initial contamination of the artificially bacon was between 4 and 5 log_10_ CFU/g for all strains inoculated singly or in a mix. The control group was treated by spraying sterile PBS without the addition of inoculum. The inoculation levels were selected according to previous studies of meat products (Bredholt et al., 2001; Alves et al., 2006). After inoculation, smoked bacon specimens were transferred to sterile plastic bags and vacuum-packaged in a sterile environment, then kept at 0–4°C for 45 days. The day upon which specimens were thawed and uninoculated was designated day 0. Analyses were carried out aseptically after 7, 15, 22, 30, and 45 days of storage, three packages from each treatment were randomly chosen at each time point.

### Measurements of pH and Total Volatile Basic Nitrogen

The pH of bacon was monitored in triplicate by using S210 SevenCompact^TM^ pH meter (Mettler-Toledo), and previously calibrated in standard solutions at pH 4.01 and 7.01 at room temperature. Total volatile basic nitrogen (TVB-N) was detected by the Kjeldahl method with an automatic Kjeldahl nitrogen analyzer (Shanghai Xianjian Instrument Co., Ltd., China). The TVB-N value (mg/100 g bacon) was calculated according to the utilization of hydrochloric acid (0.01 mol/L).

### Microbiological Analysis

For each sample, 25 g was homogenized aseptically in 225 mL cold Ringer’s solution (Oxoid) for 2 min within a separate stomacher bag. Then the suspension was diluted (1:10) with sterile distilled water to acquire the final working dilution. After shaking, 0.1 mL of each dilution was spread on selective culture media: (i) total plate count (TPC) in Plate Count Agar (PCA) (Oxoid^TM^), incubated at 30°C for 48 h, (ii) psychrophilic and psychrotrophic bacteria were also isolated in PCA at 7°C for 10 d, (iii) Violet Red Bile Glucose Agar (VRBGA) (Lang Bridge) for the cultivation of Enterobacteriaceae, incubated at 37°C for 36 h, (iv) LAB on de Man Rogosa and Sharpe (MRS) agar, incubated at 30°C for 48 h, and (v) Staphylococci were enumerated on Baird-Parker Agar (Lang Bridge), and incubated at 37°C for 48 h. The MRS and VRBGA agar plates were transported in 2.5-L anaerobic culture bags (Qingdao Hope Bio-Technology Co., Ltd., Shandong, China). The results are expressed as decimal logarithms of colony forming units per gram (log_10_ CFU/g), and the method used in this study had a lower limit of detection of 2 log_10_ CFU/g.

### DNA Extraction, Pyrosequencing, and Data Analysis

#### Total DNA Extraction

The bacteria cells were analyzed following the method previously described by Li et al. (2019). The cells pellets were obtained and used to extract the total DNA according to the manufacturer’s recommendations for use of the EZNA^®^ bacterial DNA extraction kit (Omega Bio-tek, GA, United States). DNA quality and purity were determined through spectrophotometric quantification (NanoDrop Technologies, Wilmington, DE, United States).

#### Illumina High-Throughput Sequencing

Microbial diversity was determined by amplifying and sequencing the hypervariable region V3-V4 of the bacterial 16S rRNA gene, using primers containing barcodes and PCR conditions as previously reported (Polka et al., 2015; Li et al., 2019). All PCR amplification reaction mixtures were examined using 2.0% agarose gel electrophoresis with a loading buffer (containing SYRB green) and mixed in equidense ratios and purified using the GeneJET PCR Purification Kit (Thermo Fisher Scientific). HTS was conducted on the Illumina MiSeq (Illumina, United States) according to the manufacturer’s specifications. Sequencing libraries were formed using an Ion Plus Fragment Library Kit 48 rxns (Thermo Fisher Scientific), following the manufacturer’s protocol. The library quality was assessed by using a Qubit@ 2.0 fluorometer (Thermo Fisher Scientific). The library was sequenced on an Ion S5^TM^ XL platform and 400 bp/600 bp single-end reads were generated (Novogene Bioinformatics Technology Co., Ltd., Beijing, China). Raw data from a next-generation sequencing platform were submitted to the Sequence Read Archive of National Center for Biotechnology Information (NCBI), under BioProject ID PRJNA746727.

#### Bioinformatics and Data Analysis

Single-end reads were assigned based on their unique barcodes and truncated by removing the barcodes and primer sequences. Raw sequence reads were passed through quality filtering to obtain high-quality clean reads (Martin, 2011). Operational taxonomic units (OTUs) were assigned at 97% similarity levels using Uparse software (Edgar, 2013). Alpha-diversity and beta-diversity were calculated by QIIME software (Version 1.7.0), and the similarities were analyzed by non-metric multi-dimensional scaling (NMDS) using the R package vegan. OTUs were mapped to the SILVA database and classified according to phylum, class, order, family, and genus.

#### Statistical Analysis

The data pertaining to TVB-N, pH, and viable counts are presented as the mean ± SD, and significant differences in mean values were compared by one-way analysis of variance (ANOVA) (Duncan’s multiple range), with SPSS Statistics 20.0 software (SPSS Inc., Chicago, IL, United States), while *P* < 0.05 was considered statistically significant.

## Results and Discussion

### pH and Total Volatile Basic Nitrogen

**FIGURE 1:**
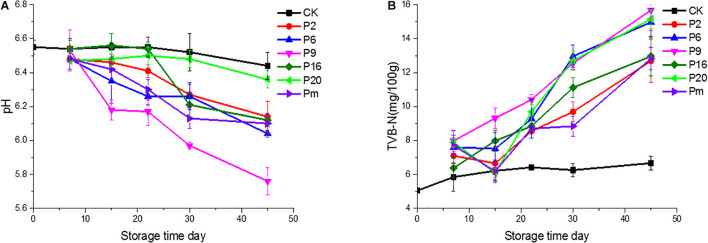
pH values and total volatile basic nitrogen (TVB-N) of bacon inoculated with potential spoilage bacteria, during refrigerated storage. The error bars were derived from the standard deviation between replicates (*n* = 3). CK, Control; P2, *Staphylococcus xylosus*; P6, *Leuconostoc mesenteroides*; P9, *Carnobacterium maltaromaticum*; P16, *Leuconostoc gelidum*; P20, *Serratia liquefaciens*; Pm, The five strains in combination.

The trend in pH changed slightly during the first 7 days among the groups shown in Figure 1. Compared with the controls, the final pH decreased significantly (*P* < 0.05) at the end of the study on day 45, reached a relatively high 6.36 for *S. liquefaciens* group, and relatively low values of 5.74 and 6.06 for *C. maltaromaticum* and *L. mesenteroides* groups, respectively. The microbial activity may lead to a significant decrease of the pH of the samples (Comi et al., 2016). Additionally, different strain groups appeared in different downtrends, after 45 days of storage, it was found that the acidification in bacon inoculated by *C. maltaromaticum* and *L. mesenteroides* was lower (*P* < 0.05) than that measured in *S. liquefaciens* and other groups. This pH reduction may be attributed to the fact that LAB rapidly became the predominant microorganism, producing lactic acid which decreased the pH and antibacterial peptide concentration (bacteriocins) (Gram et al., 2002; Kuo and Chu, 2003; Wang et al., 2013). It was probably *S. liquefaciens*, as determined by its low ability to produce the metabolites, that was associated with this acidic pH. Furthermore, the low pH may inhibit bacterial growth but the degree of inhibition varies by species. Generally, LAB and *Enterobacteriaceae* show high acid resistance and are able to grow and survive at acidic pH (Houtsma et al., 1996; Pin and Baranyi, 1998), however, *Brochothrix thermosphacta* and other species cannot grow on meat, or cause spoilage of meat under acid conditions (low pH) (Sun and Holley, 2012; Mills et al., 2014).

As one of the important chemical indicators, the change of TVB-N values may be attributed to increased protein degradation by endogenous enzymes and bacteria (Huang et al., 2014). In this study, variable production of TVB-N was observed in the different bacterial groups and showed an increasing trend. As illustrated in Figure 1, the TVB-N values (15.68 and 15.16 mg/100 g, respectively) were increased significantly (*P* < 0.05) in samples inoculated with *C. maltaromaticum* and *S. liquefaciens.* The TVB-N was relatively low 12.69 for the *S. xylosus* group, while the control samples only reached 6.66. Thus we inferred whether *C. maltaromaticum* and *S. liquefaciens*, as active producers of TVB-N, had effective spoilage potential in a manner similar to descriptions of these strains as the main spoilage organisms in spoilage species (Stohr et al., 2001; Zhang et al., 2015).

### Microbiological Analysis

Due to the different feed conditions of different bacteria, five different types of culture media that have been previously used to identify the level of microbial spoilage were used. As shown in Table 2, colonies were grown rapidly on selective culture media after incubation, and reached the lowest 7.99 log_10_ CFU/g on PCA medium at 30°C on day 15 except for the control groups. In general, all tested strains showed good growth and survival during storage, there were significant differences at the 0.05 level in final bacterial populations. In inoculated samples, the total viable count (TVC) and LAB counts increased rapidly to about 8 log_10_ CFU/g by day 15.

**TABLE 2:** Changes in viable counts of bacon inoculated with potential spoilage bacteria, during refrigerated storage after 45 days.

Colony and selective medium	Storage time (days)	Microbiological analysis (log_10_ CFU/g)						
		
		CK	P2	P6	P9	P16	P20	Pm
TVC (PCA 30°C)	0	ND	/	/	/	/	/	/
	7	ND	6.50 ± 0.66^d^	6.41 ± 0.72^c^	6.71 ± 0.47^c^	6.31 ± 0.62^c^	6.51 ± 0.44^d^	6.94 ± 0.52^c^
	15	ND	8.05 ± 0.37^c^	8.21 ± 0.73^b^	8.02 ± 0.58^b^	8.17 ± 0.71^b^	7.99 ± 0.66^c^	8.09 ± 0.82^b^
	22	ND	8.34 ± 0.84^bc^	8.72 ± 0.96^ab^	8.79 ± 0.85^ab^	8.76 ± 0.65^a^	8.37 ± 0.47^b^	8.61 ± 1.24^ab^
	30	ND	9.01 ± 1.13^a^	8.91 ± 1.05^a^	9.07 ± 1.21^a^	8.64 ± 0.84^ab^	8.66 ± 0.86^ab^	8.97 ± 0.99^a^
	45	ND	8.86 ± 0.99^b^	8.64 ± 1.12^ab^	8.74 ± 0.66^ab^	8.34 ± 0.93^ab^	8.78 ± 0.95^a^	8.72 ± 0.85^ab^
TVC (PCA 7°C)	0	ND	/	/	/	/	/	/
	7	ND	6.48 ± 1.20^d^	6.52 ± 0.46^c^	6.52 ± 0.48^c^	6.72 ± 0.59^c^	ND	6.22 ± 1.24^c^
	15	ND	8.00 ± 0.42^c^	8.04 ± 0.87^b^	7.86 ± 0.67^b^	7.55 ± 0.82^b^	5.12 ± 0.47^c^	7.74 ± 0.48^b^
	22	ND	8.21 ± 0.77^b^	8.27 ± 0.64^ab^	8.38 ± 0.57^ab^	8.62 ± 0.57^ab^	6.35 ± 0.61^b^	8.65 ± 1.08^a^
	30	ND	8.83 ± 0.94^a^	8.75 ± 0.95^a^	8.67 ± 0.91^a^	8.93 ± 1.25^a^	6.16 ± 0.79^b^	8.51 ± 1.22^ab^
	45	ND	8.61 ± 0.88^ab^	8.42 ± 0.58^ab^	8.59 ± 0.86^ab^	8.01 ± 0.76^ab^	6.58 ± 0.92^a^	8.62 ± 0.86^ab^
LAB (MRS)	0	ND	/	/	/	/	/	/
	7	ND	ND	6.37 ± 0.77^c^	ND	ND	ND	5.61 ± 0.51^e^
	15	ND	4.15 ± 0.56^c^	8.33 ± 0.59^b^	5.20 ± 0.42^c^	5.67 ± 0.47^c^	ND	5.84 ± 0.63^d^
	22	ND	3.17 ± 0.56^c^	9.06 ± 1.36^a^	8.05 ± 0.76^ab^	7.89 ± 0.58^b^	4.64 ± 0.39	6.85 ± 0.77^c^
	30	ND	7.12 ± 0.58^b^	8.88 ± 0.67^ab^	8.42 ± 0.81^a^	8.21 ± 0.67^a^	5.75 ± 0.41	7.22 ± 0.68^b^
	45	ND	7.79 ± 0.91^a^	8.74 ± 0.85^b^	7.89 ± 0.98^ab^	8.20 ± 1.17^ab^	5.39 ± 0.74	7.68 ± 0.49^a^
Staphylococcaceae (BP)	0	ND	/	/	/	/	/	/
	7	ND	3.12 ± 0.55^e^	ND	7.01 ± 0.82^c^	5.43 ± 0.92^c^	ND	5.87 ± 0.87^c^
	15	ND	4.87 ± 0.63^d^	ND	8.02 ± 0.86^b^	6.92 ± 0.43^b^	ND	7.89 ± 0.75^b^
	22	ND	5.30 ± 1.12^c^	ND	8.87 ± 1.34^ab^	7.46 ± 0.66^ab^	ND	8.46 ± 0.60^a^
	30	ND	7.48 ± 0.58^b^	ND	9.01 ± 0.68^a^	7.75 ± 0.57^a^	ND	7.84 ± 0.63^b^
	45	ND	8.21 ± 0.58^a^	ND	8.64 ± 1.24^ab^	6.07 ± 0.85^ab^	ND	8.24 ± 0.88^ab^
Enterobacteriaceae (VRBGA)	0	ND	/	/	/	/	/	/
	7	ND	5.94 ± 0.41^d^	ND	2.41 ± 0.35^d^	5.27 ± 0.38^d^	6.41 ± 0.74^d^	4.47 ± 0.57^a^
	15	ND	7.92 ± 0.87^c^	4.53 ± 0.42^c^	4.27 ± 0.48^bc^	7.72 ± 0.57^b^	7.39 ± 0.87^c^	6.47 ± 0.86^a^
	22	ND	8.32 ± 1.08^b^	8.48 ± 1.05^b^	4.65 ± 0.55^c^	7.52 ± 1.30^c^	8.22 ± 1.18^ab^	7.24 ± 1.02^a^
	30	ND	8.46 ± 0.94^a^	8.57 ± 1.24^ab^	6.92 ± 0.64^b^	8.67 ± 0.71^a^	8.16 ± 1.22^b^	8.66 ± 1.42^a^
	45	ND	8.62 ± 0.59^ab^	8.28 ± 0.86^a^	7.78 ± 0.97^a^	8.28 ± 0.99^ab^	8.49 ± 0.87^a^	8.53 ± 1.04^a^

*Means with different letters within the same column indicate a significant difference at *P* < 0.05.*

*CK, control samples; P2, *Staphylococcus xylosus*; P6, *Leuconostoc mesenteroides*; P9, *Carnobacterium maltaromaticum*; P16, *Leuconostoc gelidum*; P20, *Serratia liquefaciens*; Pm, a mixture of these five strains.*

Microorganisms interacted with each other when the order of magnitude reached levels of approximately 7–9 log_10_ CFU/g (Gram et al., 2002). In control samples, the microorganism populations were observed to be below the detection limit (2 log_10_ CFU/g) after 45 days under refrigeration, which indicated that gamma irradiation was an efficient treatment when controlling the number of microorganisms and extending the shelf-life with no adverse changes or deterioration (O’bryan et al., 2008; Chen et al., 2016). Irradiated bacon was used as a suitable host material to simulate natural cooked meat of a substantially consistent nature. Sterile bacon was considered a suitable alternative model as an initial screening procedure. *Staphylococcaceae* populations inoculated with *L. mesenteroides* and *S. liquefaciens* at lower levels (<2 log_10_ CFU/g), indicated that the growth was significantly inhibited and suppressed.

### Bacterial Richness and Diversity

After quality filtering and merging of paired reads, the total number of 7,752,153 effective sequences (tags) could be remained from 108 samples; sequence lengths were between 403 and 478 bp (Table 3). These effective sequences were clustered into 14,875 OTUs with 97% similarity level by using UPARSE algorithm embedded in Qiime. High Good’s coverage at least 99.7% suggested that most of the bacteria OTUs in samples could be captured. The α-diversity indices including observed OTUs, Chao1, and Shannon diversity index were calculated and shown as boxplots (Supplementary Figure 1). Shannon’s index represents the species diversity, the observed OTUs and Chao1 index reflected species richness. According to Supplementary Figure 1, these three indices decreased over storage time, and much higher bacterial diversity in control samples and samples at initial stage were observed compared with the middle-late stages of storage after day 15. This behavior could directly show that the richness and diversity of microorganisms had been decreased and a subset of bacteria became dominant. High Good’s coverage (≥97.3%) indicated that the majority of microbial phylotypes were well-captured.

**TABLE 3:** Alpha diversity estimation of the 16S rRNA gene libraries by sequencing on an Ion S5^TM^ XL platform in bacon.

	Sample name	Total tags	OUTs	Shannon	Simpson	Chao1	ACE	Goods coverage
**Day 0**	CK.0d	71493 ± 14839	312 ± 10	5.878 ± 0.385	0.964 ± 0.011	324.383 ± 7.477	322.247 ± 8.397	0.999
	CK.7d	68496 ± 13865	321 ± 15	5.469 ± 0.123	0.933 ± 0.016	328.667 ± 27.319	324.478 ± 20.440	0.999
	P2.7d	64053 ± 15249	239 ± 30	4.646 ± 0.916	0.885 ± 0.073	257.667 ± 40.819	258.817 ± 39.047	0.998
	P6.7d	69774 ± 11907	210 ± 61	3.774 ± 1.309	0.759 ± 0.186	237.105 ± 62.780	238.501 ± 60.792	0.998
**Day 7**	P9.7d	80138 ± 43	204 ± 9	3.893 ± 0.520	0.785 ± 0.094	237.285 ± 3.592	233.512 ± 9.352	0.998
	P16.7d	77670 ± 4315	269 ± 51	5.516 ± 0.300	0.951 ± 0.011	284.552 ± 55.676	286.245 ± 51.688	0.998
	P20.7d	60857 ± 4449	264 ± 1	5.591 ± 0.301	0.957 ± 0.014	286.079 ± 18.371	287.616 ± 13.541	0.998
	Pm.7d	62284 ± 11974	223 ± 18	4.679 ± 1.139	0.867 ± 0.118	244.855 ± 15.973	242.537 ± 16.279	0.998
**Day 15**	CK.15d	79773 ± 685	301 ± 14	5.468 ± 0.527	0.939 ± 0.039	315.205 ± 12.794	310.881 ± 13.723	0.999
	P2.15d	61970 ± 13686	165 ± 137	3.563 ± 1.482	0.804 ± 0.110	210.937 ± 131.344	227.950 ± 114.921	0.998
	P6.15d	82203 ± 3496	107 ± 100	2.789 ± 1.574	0.704 ± 0.163	127.588 ± 103.329	130.114 ± 103.902	0.999
	P9.15d	80076 ± 65	46 ± 10	1.485 ± 0.933	0.430 ± 0.294	66.277 ± 8.910	70.344 ± 18.673	0.999
	P16.15d	80117 ± 104	114 ± 57	3.209 ± 0.526	0.811 ± 0.044	145.363 ± 59.737	151.362 ± 56.029	0.998
	P20.15d	80111 ± 59	205 ± 104	4.100 ± 1.870	0.869 ± 0.092	243.200 ± 76.977	249.795 ± 75.928	0.998
	Pm.15d	61511 ± 16295	63 ± 30	3.213 ± 0.226	0.857 ± 0.041	84.118 ± 41.677	91.340 ± 46.863	0.999
**Day 22**	CK.22d	73183 ± 12127	293 ± 17	5.616 ± 0.362	0.945 ± 0.027	293.666 ± 17.897	293.666 ± 17.897	1.000
	P2.22d	79485 ± 1554	47 ± 6	2.653 ± 0.347	0.754 ± 0.076	57.783 ± 11.145	66.961 ± 14.723	0.999
	P6.22d	78300 ± 3148	57 ± 18	2.786 ± 0.071	0.785 ± 0.009	71.444 ± 20.205	74.739 ± 12.082	0.999
	P9.22d	80132 ± 68	50 ± 5	2.452 ± 0.052	0.726 ± 0.022	73.174 ± 7.476	81.311 ± 13.151	0.999
	P16.22d	80163 ± 59	46 ± 3	2.778 ± 0.224	0.787 ± 0.036	68.714 ± 5.765	84.755 ± 14.827	0.999
	P20.22d	62869 ± 6208	99 ± 77	3.229 ± 0.911	0.840 ± 0.057	122.927 ± 85.611	127.332 ± 88.602	0.998
	Pm.22d	62525 ± 15307	40 ± 12	3.072 ± 0.443	0.834 ± 0.079	47.952 ± 14.375	53.788 ± 15.813	0.999
**Day 30**	CK.30d	72405 ± 13556	345 ± 20	5.902 ± 0.522	0.960 ± 0.024	363.222 ± 11.926	355.820 ± 10.079	0.999
	P2.30d	69744 ± 9827	39 ± 5	2.643 ± 0.231	0.756 ± 0.046	58.660 ± 5.395	74.742 ± 22.268	0.999
	P6.30d	74137 ± 5241	65 ± 12	2.859 ± 0.232	0.789 ± 0.040	92.825 ± 28.131	100.961 ± 35.978	0.999
	P9.30d	80127 ± 39	41 ± 5	2.598 ± 0.097	0.753 ± 0.032	60.322 ± 9.067	71.335 ± 17.811	0.999
	P16.30d	70178 ± 14083	47 ± 5	2.852 ± 0.034	0.812 ± 0.007	58.403 ± 2.984	65.936 ± 5.569	0.999
	P20.30d	58211 ± 6360	52 ± 27	2.739 ± 2.516	0.805 ± 0.013	78.322 ± 43.259	76.269 ± 32.442	0.999
	Pm.30d	60813 ± 16677	40 ± 14	3.183 ± 0.175	0.858 ± 0.025	54.166 ± 21.391	61.578 ± 27.871	0.999
**Day 45**	CK.45d	80115 ± 32	328 ± 6	5.711 ± 0.296	0.953 ± 0.013	346.329 ± 17.584	338.845 ± 9.457	0.999
	P2.45d	72141 ± 2356	42 ± 2	2.933 ± 0.112	0.810 ± 0.019	57.400 ± 12.770	63.965 ± 15.422	0.999
	P6.45d	76042 ± 7160	51 ± 2	2.883 ± 0.079	0.805 ± 0.014	76.375 ± 5.188	83.236 ± 6.265	0.999
	P9.45d	80108 ± 42.158	52 ± 6	2.145 ± 1.109	0.599 ± 0.329	91.277 ± 48.990	91.281 ± 33.496	0.999
	P16.45d	59889 ± 6463	66 ± 10	2.915 ± 0.137	0.819 ± 0.014	83.504 ± 10.886	87.552 ± 15.329	0.999
	P20.45d	64865 ± 7519	47 ± 11	2.548 ± 0.054	0.788 ± 0.009	65.441 ± 8.010	77.858 ± 13.622	0.999
	Pm.45d	60880 ± 16652	56 ± 4	3.309 ± 0.148	0.873 ± 0.018	95.923 ± 22.901	98.431 ± 13.871	0.999

*CK, control samples; P2, *Staphylococcus xylosus*; P6, *Leuconostoc mesenteroides*; P9, *Carnobacterium maltaromaticum*; P16, *Leuconostoc gelidum*; P20, *Serratia liquefaciens*; Pm, a mixture of these five strains.*

### Composition of Bacterial Community

Bacterial community variations during bacon storage revealed by 16S rRNA gene and the mean relative abundance of microbial phyla are shown in Supplementary Figure 2. A total of 21 identified phyla were observed, and *Proteobacteria* and *Firmicutes* were the dominant phyla in the microbiota, representing 78.98–99.97% of the total catch. The proportion was associated with inoculation, for example, after inoculation with *S. liquefaciens*, *Proteobacteria* continuously increased and *Firmicutes* decreased, the highest *Firmicutes* level (99.97%) was reached at day 45, and also showed a much higher percentage (96.64%) at the genus level of *Serratia.* After inoculation with *C. maltaromaticum* (P9), after 45 days of storage, *Firmicutes* increased up to 98.43% on day 15, then decreased to 77.45% on day 15, while *Serratia* increased to 22.51% of the relative total abundance of *Proteobacteria.* The vast majority of the top 100 genera belong to the families *Firmicutes* and *Proteobacteria* (Supplementary Figure 3).

**FIGURE 2:**
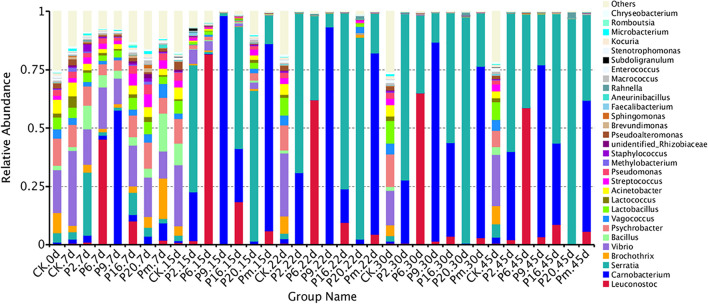
Dynamics in relative abundance (%) of bacterial taxa at the phylum level in bacon inoculated with potential spoilage bacteria. Each bar represents the relative abundance of each group, below the top-30 abundances (at genus level) data were merged. CK, Control; P2, *Staphylococcus xylosus*; P6, *Leuconostoc mesenteroides*; P9, *Carnobacterium maltaromaticum*; P16, *Leuconostoc gelidum*; P20, *Serratia liquefaciens*; Pm, The five strains in combination.

**FIGURE 3:**
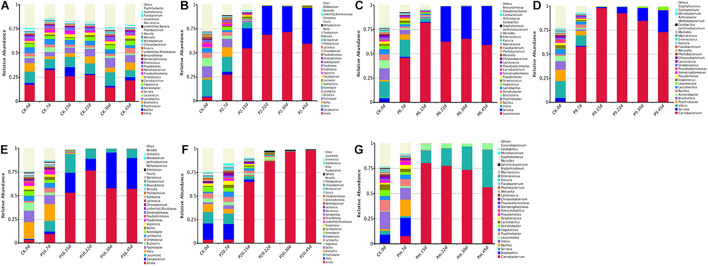
Dynamics in relative abundance (%) of bacterial taxa at the genus level in bacon inoculated with potential spoilage bacteria. Each bar represents the relative abundance of each group, below the top 30 abundance (at genus level) data were merged. CK, Control; P2, *Staphylococcus xylosus*; P6, *Leuconostoc mesenteroides*; P9, *Carnobacterium maltaromaticum*; P16, *Leuconostoc gelidum*; P20, *Serratia liquefaciens*; Pm, The five strains in combination.

For a more detailed analysis, bacterial community dynamics at the genus level were studied, and a total of 367 bacteria identified were summarized in Figure 2. Control samples had the highest number of OTUs (Table 3) and the lowest loads (those below the detection limit) throughout the storage period, implying more bacterial diversity and relative stability. As shown in Figure 3A, the main microbial groups were represented by *Vibrio* (23.38%), *Psychrobacter* (10.61%), *Lactobacillus* (6.67%), *Brochothrix* (5.88%), *Acinetobacter* (4.85%), *Serratia* (3.27%), *Bacillus* (3.17%), and *Pseudomonas* (2.54%). Similar microbial loads were frequently observed in processed meat products in the initial stage of storage (Chaillou et al., 2015; Quijada et al., 2018; Juárez-Castelán et al., 2019), indicating that these bacteria were primarily derived from raw meat and the processing unit (Borch et al., 1996; Hu et al., 2008; Chaillou et al., 2015). Therefore, these observations confirmed that irradiation could delay microbial growth and suppress final counts of spoilage microorganisms, extending the shelf-life stability of bacon.

Microbial communities evolved from day 7 to day 45 in bacon with inoculation during the storage process, where the microbial composition became less diverse and apparently more stable, with significantly decreased Chao 1 and Shannon diversity indices (Table 3 and Supplementary Figure 1). Furthermore, *Serratia*, *Carnobacterium*, and *Leuconostoc* dominated at the end of the storage, suggesting that these members had strong survival rates and competitiveness.

The bacterial community of the various inoculations significantly differed from each other at the genus level. According to Figure 3F, a rapidly increased percentage of *Serratia* to 96.64% dominated the microbial population, after the samples were inoculated with *S. liquefaciens*, during 45 days of storage. Among the *Enterobacteriaceae* family, *Serratia* spp. are the most commonly found genus in meat products and often contribute to spoilage (Doulgeraki et al., 2011, 2012). *Serratia liquefaciens* was found in high numbers after refrigerated storage of packages of minced meat, such that they actually could spoil the product (Lindberg et al., 1998), and comprised one of the most common species of *Enterobacteria* in spoiled hams (Paarup et al., 1999; Losantos et al., 2000), where they typically reached 5–7 log_10_ CFU/g (Gram et al., 1999). In this study, Figure 3C illustrates that the *Serratia* populations grew rapidly and reached a peak to become dominant on day 15, then reached their stationary phase and suppressed the growth of other spoilage organisms. *Serratia* spp. may produce several antimicrobial metabolites, which have been characterized as strong N-acyl-homoserine lactone (AHL) producers in meat (Bruhn et al., 2004). They may use quorum sensing to monitor their population density, synchronize their physiological functions, and socially interact with other bacteria (Van Houdt et al., 2007), thus we anticipated that once *Serratia* reached maximum abundance, it could make an important contribution to other spoilage-related bacteria.

The samples inoculated with *C. maltaromaticum* and *L. mesenteroides* underwent a more complex composting process of bacterial community succession. *Carnobacterium* and *Leuconostoc* increased sharply after inoculation and reached a maximum relative abundance of 97.95 and 81.6% at day 15 (Figures 3C,D), respectively: however, the proportions thereof decreased to 73.60 and 58.67% at day 45, remaining in relatively high abundance and accompanied with a rapid increase of *Serratia*. *Carnobacterium maltaromaticum* inoculated with sterile sliced beef, then grew well and achieved maximum population after 2–8 weeks (Leisner et al., 1995). *Carnobacterium* spp. are ubiquitous psychrotrophic LAB that could grow in a wide variety of meat products at lower temperatures, and are commonly predominant members of the microflora, which may be conducive to rapid deterioration during storage (Leisner et al., 2007; Casaburi et al., 2011). *Carnobacteria* possessed the capability to produce antimicrobial peptides, bacteriocins, and wide spectrum action against pathogenic and spoilage bacteria (Leisner et al., 2007). *Leuconostoc mesenteroides* was the dominant species and consequently was responsible for the spoilage of commercial bacon (Comi et al., 2016). Morcilla de Burgos inoculated with *L. mesenteroides*, as a new species, grew more rapidly and influenced the signs of spoilage due to its more energy-efficient metabolism (Diez et al., 2009). Kotzekidou and Bloukas (1998) reported that inoculation of *Lactobacillus alimentarius* in vacuum-packed frankfurter-type sausage could increase LAB populations and suppress other saprophytic microorganisms.

Lactic acid bacteria produce various antimicrobial components, such as organic acids, hydrogen peroxide, ethanol, bacteriocins, and other substances (Holzapfel et al., 1995; Drosinos et al., 2006), one or more these metabolites refer to the inhibition of other undesirable bacteria, including spoilage microorganisms and pathogenic bacteria (Metaxopoulos et al., 2002; Drosinos et al., 2006). In this study, *Carnobacterium* and *Leuconostoc* reached maximum relative abundance on day 15, thereafter the suppression of *Serratia* by them was slowly alleviated (Figures 3C,D), which could be explained by the fact that the antimicrobial metabolites from lactic acid strains were ineffective as a mechanism of control.

The genera that presented the greatest abundances of samples inoculated with *S. xylosus* (P2) were *Serratia* (59.26%) and *Carnobacterium* (37.76%) on day 45 (Figure 3B), while the *Staphylococcus* was found at a very low level (0.01%). Similarly, the prevalent species were also *Serratia* (55.37%) and *Carnobacterium* (34.84%) inoculated with *L. gelidum* (P16), containing only a small proportion of *Leuconostoc* (8.7%) (Figure 3E). The bacterial load in the bacon product inoculated with *S. xylosus* and *L. gelidum*, increased initially, then decreased, indicating antimicrobial substances may be generated by the predominant *Serratia* and *Carnobacterium* (probably AHLs and bacteriocins). *Serratia* and *Carnobacterium* gradually outcompeted all other bacteria and became the dominant species with increasing storage time, which was consistent with the results from the viable counts. *Staphylococcus xylosus* and *L. gelidum* displayed a slower growth of the species and were deemed less competitive among microorganisms.

*Carnobacteria* had shown the ability to produce metabolites with antimicrobial activity (peptides and bacteriocins) and could inhibit spoilage bacteria, such as LAB and *Enterococcus* (Leisner et al., 2007; Doulgeraki et al., 2012). Hydrogen peroxide and lactic acid are produced by a number of LAB (e.g., *Carnobacteria* spp. and *Leuconostoc* spp.), *Staphylococcus* spp. are more sensitive to these than most LAB, and would either be inhibited or destroyed (Holzapfel et al., 1995). *Leuconostoc gelidum* often prevail in chilled-stored, packaged, nutrient-rich, foods, such as cooked meats (Säde, 2011), however, *L. gelidum* cannot obtain energy from glycogen, proteinaceous substrates, lactate, or fatty acids, and its growth is thus inhibited. Additionally, *Carnobacterium* (56.16%) and *Serratia* (36.74%) grew rapidly and accounted for the most part of the microbial community in sterile bacon specimens inoculated with the five mixed strains (Pm) on day 45 (Figure 3G), and the proportion of *Leuconostoc* was only 5.73%. Referring to these results, the growth of *Leuconostoc* may also be inhibited by the strains of *Serratia* and *Carnobacterium.* Compared with the *Serratia*, the occurrence of *Carnobacterium* usually reported in meats and dairy products is most often ignored. Since MRS agar commonly used for LAB enumeration contained acetate it was efficient in terms of inhibiting *Carnobacterium* growth.

#### Microbial Diversity and Changes During Storage

**FIGURE 4:**
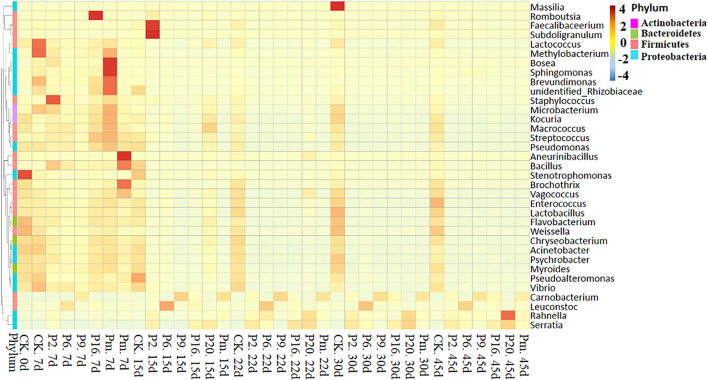
Heatmap showing the changes in the microbial communities of bacon inoculated with potential spoilage bacteria. The group heatmaps represent a homogenization of numerical values of corresponding points. CK, Control; P2, *Staphylococcus xylosus*; P6, *Leuconostoc mesenteroides*; P9, *Carnobacterium maltaromaticum*; P16, *Leuconostoc gelidum*; P20, *Serratia liquefaciens*; Pm, The five strains in combination.

To investigate the succession in microbial communities of bacon inoculated with spoilage bacteria during storage, heatmaps of bacteria at the genus level phylotypes were plotted (Figure 4 and Supplementary Figure 4). Among the generated heatmaps, the redder color denotes higher relative abundances, and the greener color represents lower abundances. According to Supplementary Figure 4, a relatively high diversity was observed in control samples throughout the storage period, due to the low concentration of viable counts (Table 2), showing that they should form relatively stable compositions. At 7 days of storage after inoculation with spoilage bacteria, the microbial community composition differed among different groups and indicated less diversity in terms of trend. During the late storage period, *Serratia*, *Carnobacterium*, and *Leuconostoc* were clustered with the highest abundance in bacon inoculated with different bacterial strains, implying that the microbial communities became minimally diverse. Similar results were found by Nychas et al. (2008), who found the gradually stable microflora composition in the late stage of storage was generally the predominant bacteria. Additionally, the more abundant dominant bacterium, the less diverse the microbial community in the late storage stages, and the specimens inoculated with such strains evidently reduced the abundance of the reference microorganisms.

**FIGURE 5:**
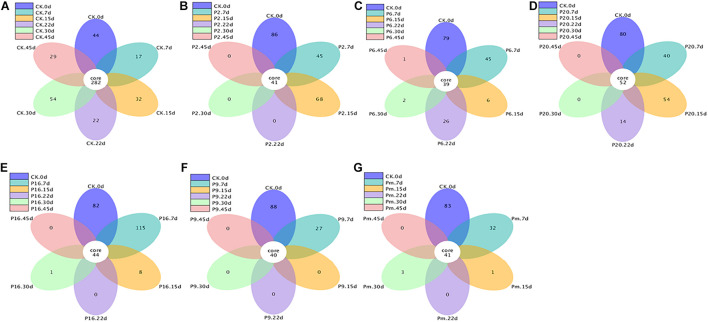
Flower figure showing the unique and shared OTUs of the bacterial communities across different stages. Only with more than five groups can the flower figure be shown. The plotting was carried out after homogenization of all samples. CK, Control; P2, *Staphylococcus xylosus*; P6, *Leuconostoc mesenteroides*; P9, *Carnobacterium maltaromaticum*; P16, *Leuconostoc gelidum*; P20, *Serratia liquefaciens*; Pm, The five strains in combination.

Similarities and differences among the groups were also analyzed using flower plots (Figure 5). According to Figure 5, for control samples, 282 groups (the largest percentage of all groups) were classified as core OTUs. Besides core OTUs, there were 44, 17, 32, 22, 54, and 29 unique OTUs on days 0, 7, 15, 22, 30, and 45, the number remained stable until the end of the storage period, indicating that the microbial composition changed slightly in control samples. In the inoculated groups, the core OTUs (39–42) were significantly lower than the control groups, and unique OTUs on days 7 and 15 were relatively large, then very few were available, intimating that the bacterial community composition changed significantly. In conclusion, the microbiota changed toward simpler and similar communities with increasing storage time.

**FIGURE 6:**
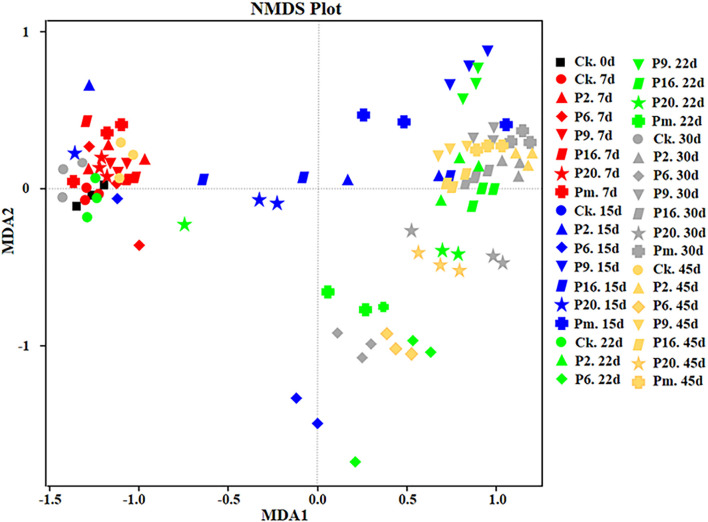
Non-metric multi-dimensional scaling analysis of the bacterial community structure of bacon inoculated with potential spoilage bacteria. The analysis was based on the relative abundances of all genera of microbiota detected by high-throughput sequencing. CK, Control; P2, *Staphylococcus xylosus*; P6, *Leuconostoc mesenteroides*; P9, *Carnobacterium maltaromaticum*; P16, *Leuconostoc gelidum*; P20, *Serratia liquefaciens*; Pm, The five strains in combination.

Additionally, non-metric multidimensional scaling (NMDS) analysis was performed to compare the differences and similarity of the community composition data (Figure 6). The figure shows joint-plot NMDS maps illustrating the bacterial community structure and the successive and dynamic changes prolonging the storage period. Figure 6 demonstrates the continuing shifts (above plane to below plane) from the primary stable period (until day 7) to a late state (until day 45). The control groups and inoculated groups on day 7 showed similar microbiota forming one cluster, then scattered, which suggested that the similarity and differences were present among the groups and that the microbiota was affected by storage time.

Foods can support a complex microflora and interactions between different species of microorganisms. In this study, the inoculation decreased the levels of potential microorganisms and inhibited the growth of numerous organisms that correlate with meat spoilage. In general, high bacterial diversities were observed in the early stage of storage while *Serratia*, *Carnobacterium*, and *Leuconostoc* became the most abundant genera with prolonged storage. We can conclude only that the reason for the dominance is not exclusively their rapid growth; it cannot be that other elements acted as competing organisms since they were dominant. Gram (1993) found that the bacterial selection in the microflora of food products is the influence of microbial interactions.

In food ecosystems, the interactions between microbial growth and enzyme activities have been shown to cause various consequences such as: growth promotion, symbiotic relationships, growth inhibition, and competition (Gram et al., 2002; Andreevskaya et al., 2018). LAB-meat interactions have been studied frequently: in vacuum-packed and refrigerated meat products, it is reported that spoilage arises from the interaction among LAB (the dominant flora) and *Enterobacteriaceae*, *pseudomonads*, *Brochothrix thermosphacta*, and other species (found in lower numbers) (Borch et al., 1996; Metaxopoulos et al., 2002; Bruhn et al., 2004). LAB could produce antimicrobial substances including organic acids and bacteriocins, which usually inhibit the growth of other microorganisms (Cleveland et al., 2001; Wang et al., 2016). *Leuconostoc mesenteroides* and *Lactobacillus curvatus* can produce bacteriocins and inhibit other spoilage microorganisms or even pathogens (Metaxopoulos et al., 2002). *Enterobacteriaceae*, notably *Serratia* spp. and *Hafnia alvei*, due to their ability to frequently become dominant in spoilage flora (Borch et al., 1996), were found to contribute to vacuum-packed meat spoilage through the quorum sensors (QS) systems (AHLs); however, AHL-producing *Hafnia alvei* might influence the spoilage in which other organisms participated with the spoilage process (Bruhn et al., 2004).

Bacterial interactions and competition have been extensively studied for the past few decades. A variety of interactions (stimulation, delay, complete inhibition of growth, and no effects between them) could occur when lactic acid starters and probiotic bacteria were mixed (Vinderola et al., 2002). Co-culture studies can verify that interactions between *Lactobacillus sakei* 10A, *Lactobacillus sakei* LS5, and *Brochothrix thermosphacta* BT1 occurred in cooked meat (Vermeiren et al., 2006). Borch et al. (1996) found that sterilized beef inoculated with *Hafnia alwi* together with LAB, gave rise to unpleasant and unacceptable off-odors after 8 weeks, whereas no off-odors were detected with single *Hafnia alwi* culture. Inoculation with the three mixture of *Shewanella putrefaciens*, *Photobacterium phosphoreum*, and *Aeromonas* sp. in cold-smoked salmon cannot caused spoilage whereas co-inoculation of two bacteria *Brochothrix thermosphacta* and *Carnobacterium piscicola* was capable of producing off-odors (Joffraud et al., 2001). Morcilla de Burgos inoculated with *L. mesenteroid*es and *W. viridescens*, both jointly and separately cultured, particular signs of spoilage increased compared to single-cell cultures (Diez et al., 2009). A number of these studies showed that the off-odor may originate from interactions among several bacteria. Microbial spoilage was caused by the growth and reproduction of a diversity of microorganisms, two or more microbial species exchange metabolites or nutrients to cause spoilage and disrupt product interactions (Gram et al., 2002). No single *S. liquefaciens* could be identified as the cause of spoilage, the growth and activity of bacteria usually contained a mixture of species/groups (Gram et al., 1999).

To develop novel preservation technologies and develop models for predictive microbiology, an insight into understanding of microbiota, and the dynamic changes and interactions during the refrigerated storage of meat products is of great importance, however, little information regarding possible interactions responsible for meat spoilage is available, so further research is needed.

## Conclusion

In this study, the dynamic changes in bacterial community structures during the storage of bacon which had been previously inoculated with five potential spoilage bacteria, were evaluated. Using HTS, 21 phyla, and 367 bacteria genera were identified, with the control samples exhibiting the highest microbial diversity. Compared with the other groups, major microbiological and physicochemical changes appeared after 15 days, with the changes becoming gradually stable and less diverse bacterial communities appearing in the later stages of the storage period. *Serratia liquefaciens*, *C. maltaromaticum*, and *L. mesenteroides* were found to be more competitive species. The results from this study provide a basic understanding of the microbial composition and changes in the bacterial profile of bacon during the spoilage process. Although further investigations are needed to increase our understanding of the interactions between the microbial communities within the spoilage environment, it is expected that this study will be of benefit to further improve the shelf-life of meat products.

## Data Availability Statement

The datasets presented in this study can be found in online repositories. The names of the repository/repositories and accession number(s) can be found below: NCBI (accession: PRJNA746727).

## Author Contributions

YS and BX designed the experiments. XL carried out the experiments. QX analyzed the experimental results and assisted with the Illumina sequencing. HZ analyzed the sequencing data and developed the analysis tools. XL wrote the manuscript. All authors contributed to the article and approved the submitted version.

## Conflict of Interest

The authors declare that the research was conducted in the absence of any commercial or financial relationships that could be construed as a potential conflict of interest.

## Publisher’s Note

All claims expressed in this article are solely those of the authors and do not necessarily represent those of their affiliated organizations, or those of the publisher, the editors and the reviewers. Any product that may be evaluated in this article, or claim that may be made by its manufacturer, is not guaranteed or endorsed by the publisher.
