# Supplementary figures and images for: Analysis of Microbial Diversity and Dynamics During Bacon Storage Inoculated With Potential Spoilage Bacteria by High-Throughput Sequencing
Source: Front Microbiol. 2021 Sep 28;12:713513. doi: 10.3389/fmicb.2021.713513 (PMC8506151; doi:10.3389/fmicb.2021.713513)

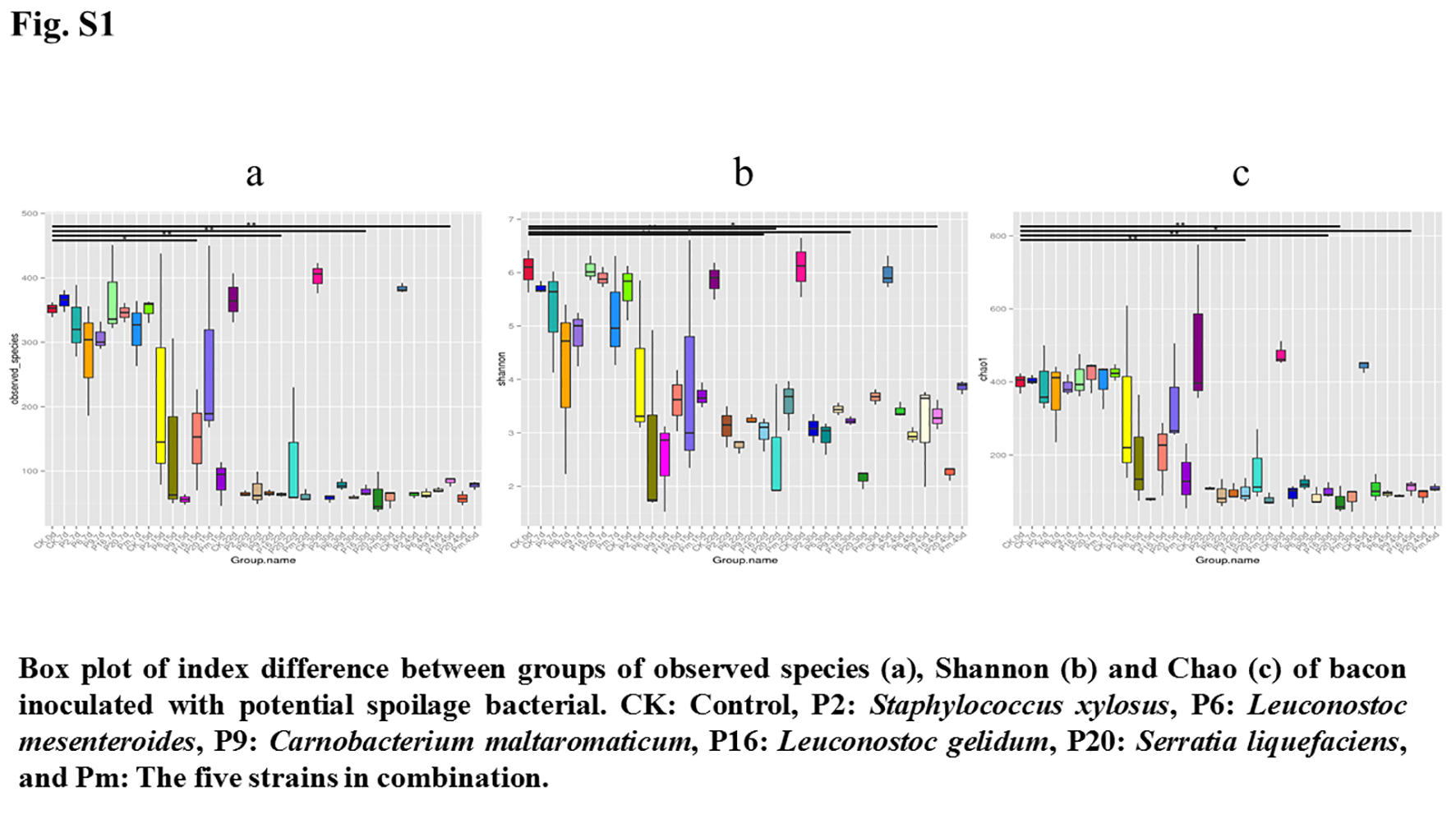

Supplement: Supplementary file 1 [file Image_1.TIF]

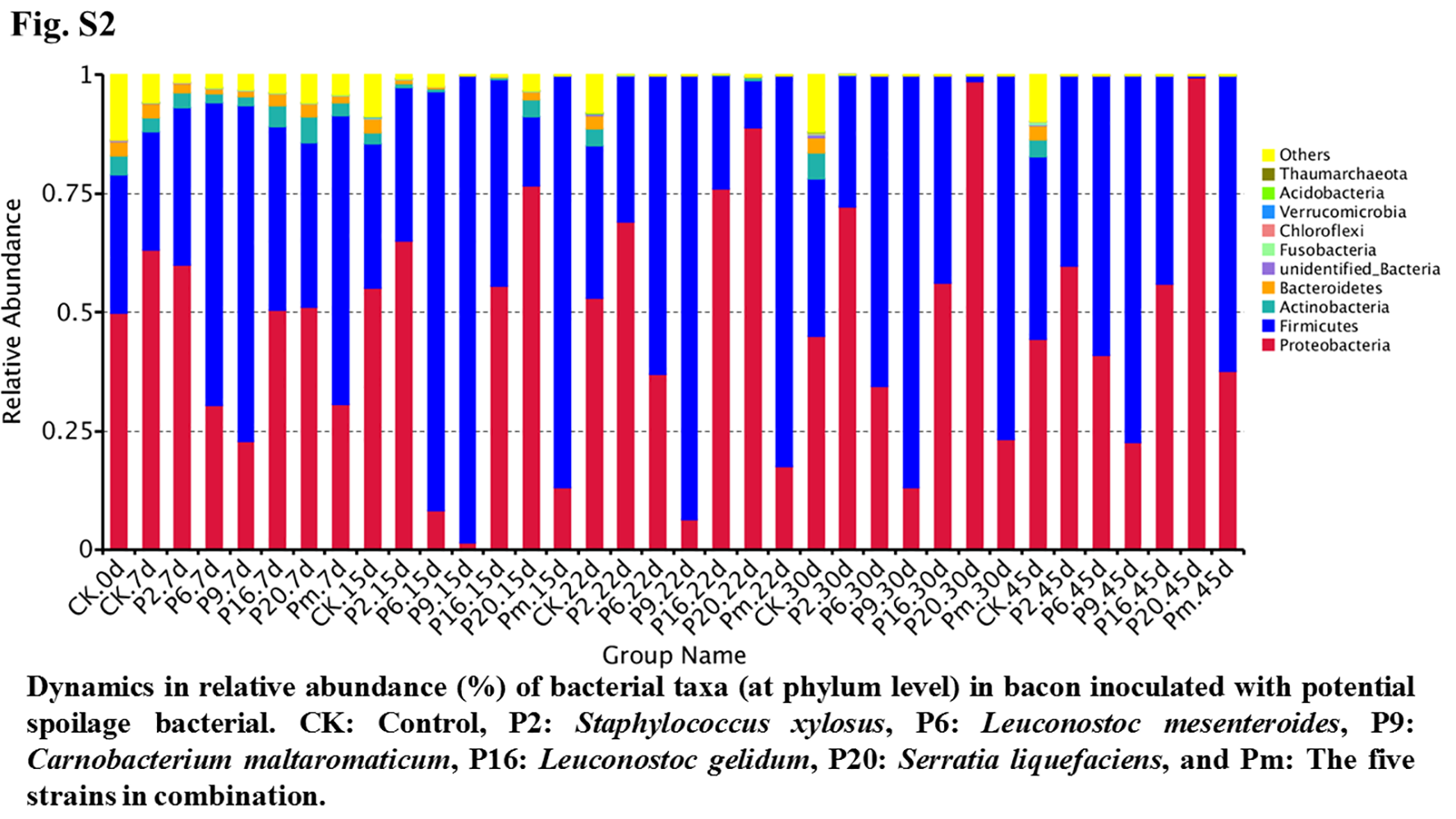

Supplement: Supplementary file 2 [file Image_2.TIF]

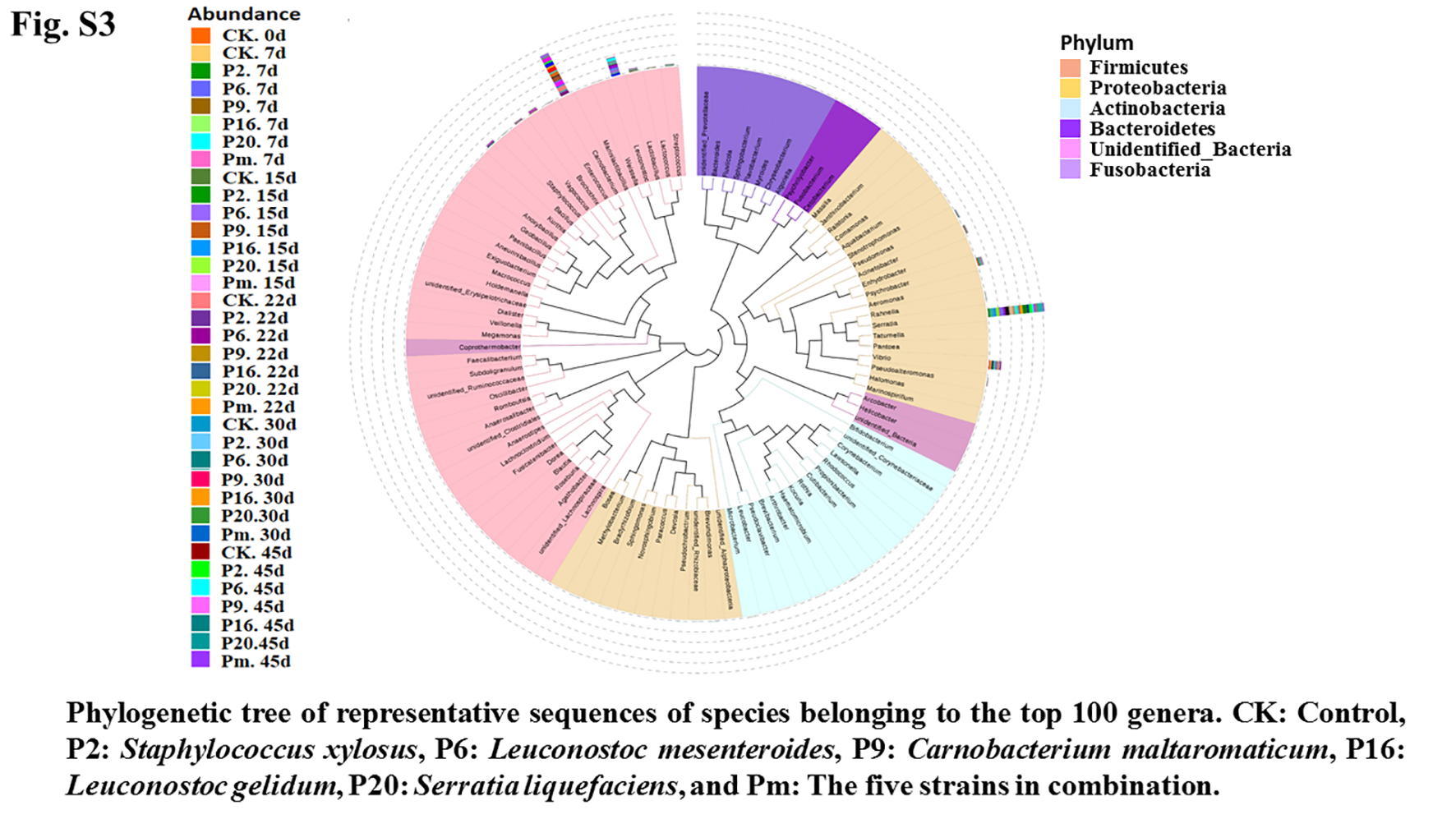

Supplement: Supplementary file 3 [file Image_3.TIF]

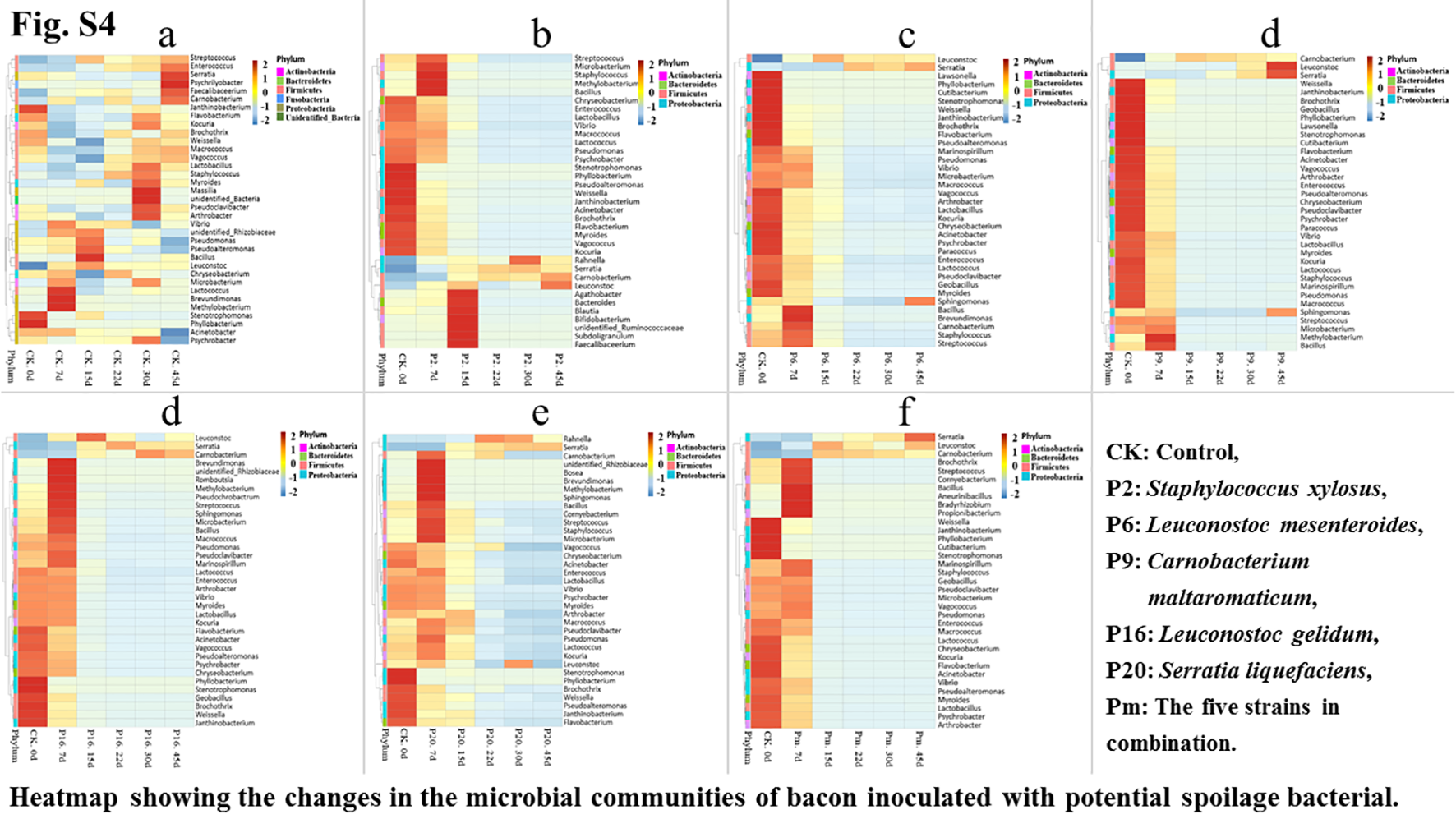

Supplement: Supplementary file 4 [file Image_4.TIF]
